# Two wavelength band emission WGM lasers via photo-isomerization

**DOI:** 10.1515/nanoph-2023-0522

**Published:** 2023-10-20

**Authors:** Kun Ge, Jun Ruan, Ningning Liang, Dan Guo, Libin Cui, Naeem Iqbal, Tianrui Zhai

**Affiliations:** Faculty of Science, Beijing University of Technology, Beijing, 100124, China

**Keywords:** two wavelength band emission, WGM laser, photo-isomerization, ESIPT

## Abstract

Wavelength switchable microcavity is indispensable component for various integrated photonic devices. However, achieving two wavelength band emission of the whispering gallery mode (WGM) laser is challenging. Here, we propose a strategy to realize two wavelength band emission WGM lasers activated by photo-isomerization based on excited-state intramolecular proton transfer (ESIPT) process in isolated/coupled polymer microfiber cavities. The WGM microcavity is built by highly polarized organic intramolecular charge-transfer (ICT) dye molecules. The two cooperative gain states of ICT dye molecules can be controlled by optimizing energy levels. Thereby, the lasing wavelength can be reversibly switched under photo-isomerization activated in the ESIPT energy-level progress. The photonic bar code can be generated by following the strategy of proposed design. This work provides a promising route to achieve switchable WGM laser in on-chip photonic integration.

## 1 Introduction

Whispering gallery mode (WGM) lasers with small mode volume and high quality (*Q*) factor, have received great interest in basic research [1], [2], [3], and applications including integrated communication devices [4], [5], [6], sensitivity detectors [7], [8], [9], chaotic optical cavity and nonlinear optical cavity [10], [11], [12], chemical and biological sensing [13], [14], [15], photonic barcodes, and reliable encryption system [16], [17], [18], [19]. The two wavelength band emission WGM laser is a key to producing compact and multifunctional on-chip integrated optoelectronic devices. However, it is difficult to achieve a wide range of two wavelength band emission WGM laser due to the energy levels of most gain materials are fixed. In general, the switchable lasing wavelength can be achieved through using external stimuli, such as chemical gas and solution [20, 21], temperature [22], [23], [24], tension, and compression [25]. Most of these lasers are achieved relying on variation in the effective refractive index. And these wavelength tuning laser devices remain limited due to the small range of effective refractive index modulation. Moreover, the laser performances would be inevitably degraded with external stimuli as well, which greatly influenced their wide range of demanding applications. In view of these, the tunable WGM laser is obtained by changing the concentration of dye materials along with optical parameters. Importantly, in some dye materials, a small Stokes shift is induced owing to re-absorption phenomenon, which is not conducive to the development of low threshold laser devices [26, 27].

As a typical active materials, intramolecular charge-transfer (ICT) dye molecules exhibit unique multiple molecular excited states, including trans (*E*) state, and cis (*Z*) state, which makes the dye an ideal candidate as a material for manufacturing the microcavities [28], [29], [30]. Additionally, it also possesses the unique characteristics of photo-isomerization, which can facilitate in developing the reversible two wavelength band emission WGM lasers.

Here, we propose a strategy to realize two wavelength band emission WGM lasers by exploring the phenomenon of photo-isomerization. The WGM microcavity is constructed by using the highly polarized organic ICT dye molecules as gain material exhibiting two cooperative gain states via optimizing the energy levels with advantageous of selecting excitation gain regions. The ICT dye molecules can be transferred from E-state to Z-state activated by following the principle of photo-isomerization, which enables to cultivate a dynamically switchable wide wavelength range lasing device. Thereby, the lasing wavelength can be reversibly switched in excited-state intramolecular proton transfer (ESIPT) process under photo-isomerization [31–36]. Importantly, it is worth noting that simultaneous variation in effective refractive index and ESIPT energy-level process in coupled microfiber cavity provided a switchable single mode lasing exhibiting mode moving and mode hopping like features, respectively. Finally, we applied this switchable WGM microcavity obeying photo-isomerization phenomenon to develop photonic barcodes by exploring a reliable encryption system.

## 2 Results

A typical highly polarized photo-isomerization light-emitting dye molecule disodium 4, 4′-Bis (2-sulfonatostyryl) biphenyl (S420, D-36543, Tianjin Heowns Biochem LLC, Tianjin, China) acts as chemical reagent, and retains two excited gain states such as trans-S420 state and cis-S420 state through optimizing energy levels. Polyvinyl alcohol (PVA) material exhibiting a high compatibility is selected as polymer material along with S420 due to its excellent tendency of optical transparency. Sodium dodecyl sulfate (SDS) is regarded as surfactant, and can be positively charged when dissolved in water. All these three materials (S420, PVA, SDS) are simply mixed with solvent deionized water. The concentration of gain active (S420), PVA and SDS are10 mg/mL, 16 wt% and 5 wt%, respectively. The mixed solution was heated at 70 °C for 12 h in a heating box. The polymer compound microfiber was fabricated by bottom-up pulling technology using the taper needle.

To investigate the polar solvents influence on fluorescence spectra of highly polarized organic ICT dye molecules, we produced the ICT dye solutions in different solvents with concentration of 10 mg/mL. It is noticed that emission wavelength peak is red-shifted with increasing solvent polarity (in Figure S1) which is the typical property of ICT compounds dye material [37]. The S420 can be a promising ideal material for achieving multiwavelength switchable WGM laser as apparent from its photoluminescence (PL) spectra.

**Figure 1:**
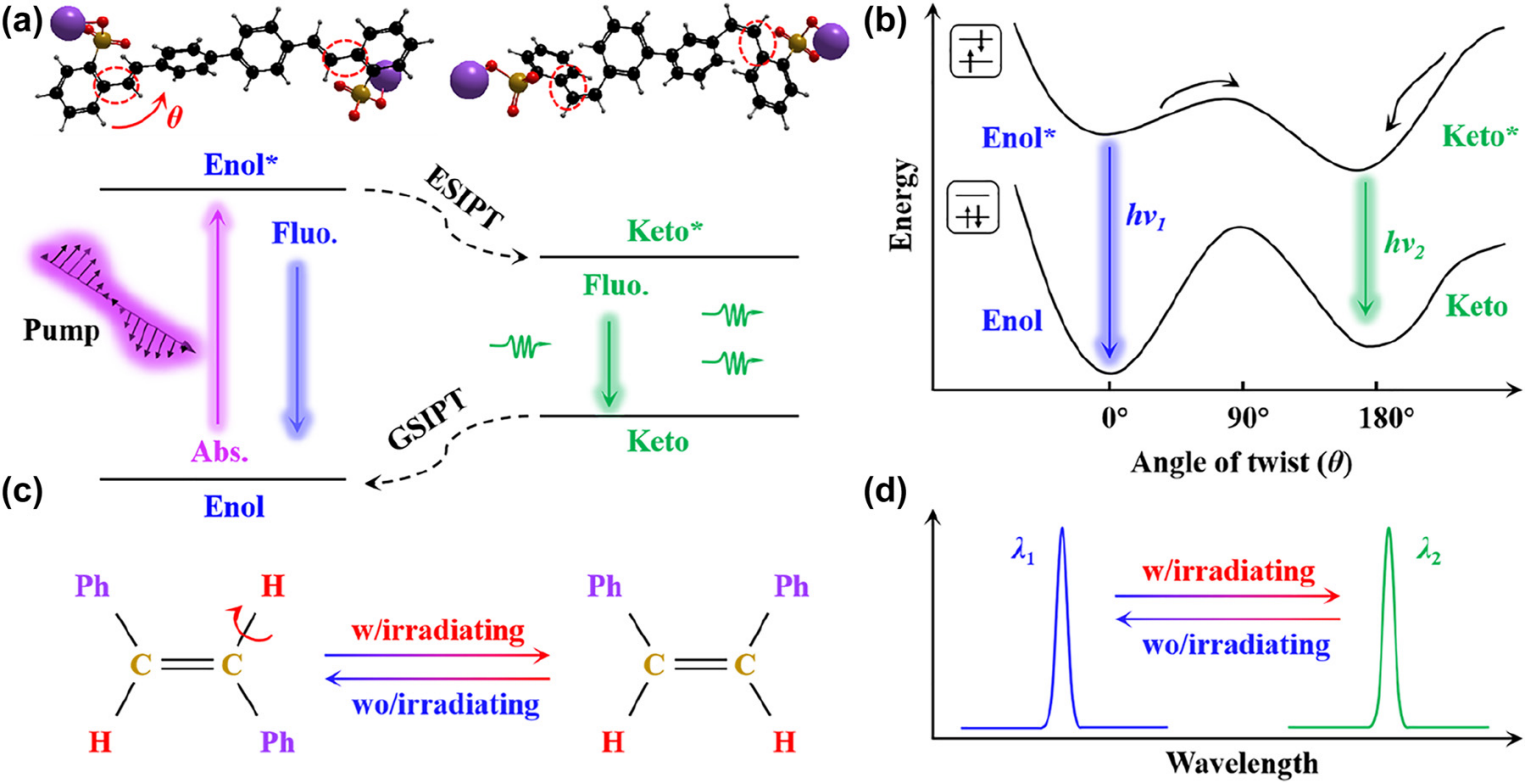
Schematic illustration of switchable lasing is based on ESIPT progress. (a) Schematic representation of ESIPT photocycle progress of ICT dye molecules in microfiber cavity. GSIPT: ground-state intramolecular proton transfer. (b) The relationship between energy and twisting angle (*θ*) for typical organic ICT dye molecules materials. (c) Schematic diagram of the conversion process of ICT dye molecules with irradiating. (d) Illustration of switchable WGM laser w/irradiating and wo/irradiating. w: with; wo: without.

The ground-state ICT dye molecules exist as cis-enol (Enol) form as shown in Figure 1a. When pumped by pulsed laser excitation, the excited Enol (Enol*) state rapidly transits to the cis-form of excited Keto (Keto*) state via the intramolecular proton transfer under photo-isomerization. The excited state proton transfer follows an immediate radiative transition from Keto* to Keto, and finally the rapid ground-state from Keto to Enol undergoes via reverse proton transfer, which thereby builds a fast four-level photocycle (Enol-Enol*-Keto*-Keto-Enol) [37].

Generally, two states laser emission is not possible to occur without considering the external stimuli. Figure 1b provides the relationship between energy and twisting angle for typical organic ICT laser dye. The trans-S420 state in the ground state (*S*_0_) represented by two squares the ground state and excited state in the upper left in Figure 1b needs to cross a very high energy barrier to reach the excited intermediate state (at twist angle 90°) as shown in Figure 1b. However, the energy barrier for trans-S420 in the excited state (*S*_1_) to reach the excited intermediate state is greatly reduced (twist angle 90°). Moreover, the process from the excited enol state to the excited ketone state is achieved by photo-isomerization. Figure 1c demonstrates the schematic diagram of the process of ICT dye molecules transitioning form trans-S420 state to cis-S420 state under irradiation. And, it will undergo reverse evolution under considering the situation of without irradiation. Finally, the lasing emission spectra can be switched from *λ*_1_ (trans-S420 state) to *λ*_2_ (cis-S420 state) as a result of photo-isomerization as shown in Figure 1d.

## 3 Discussion

**Figure 2:**
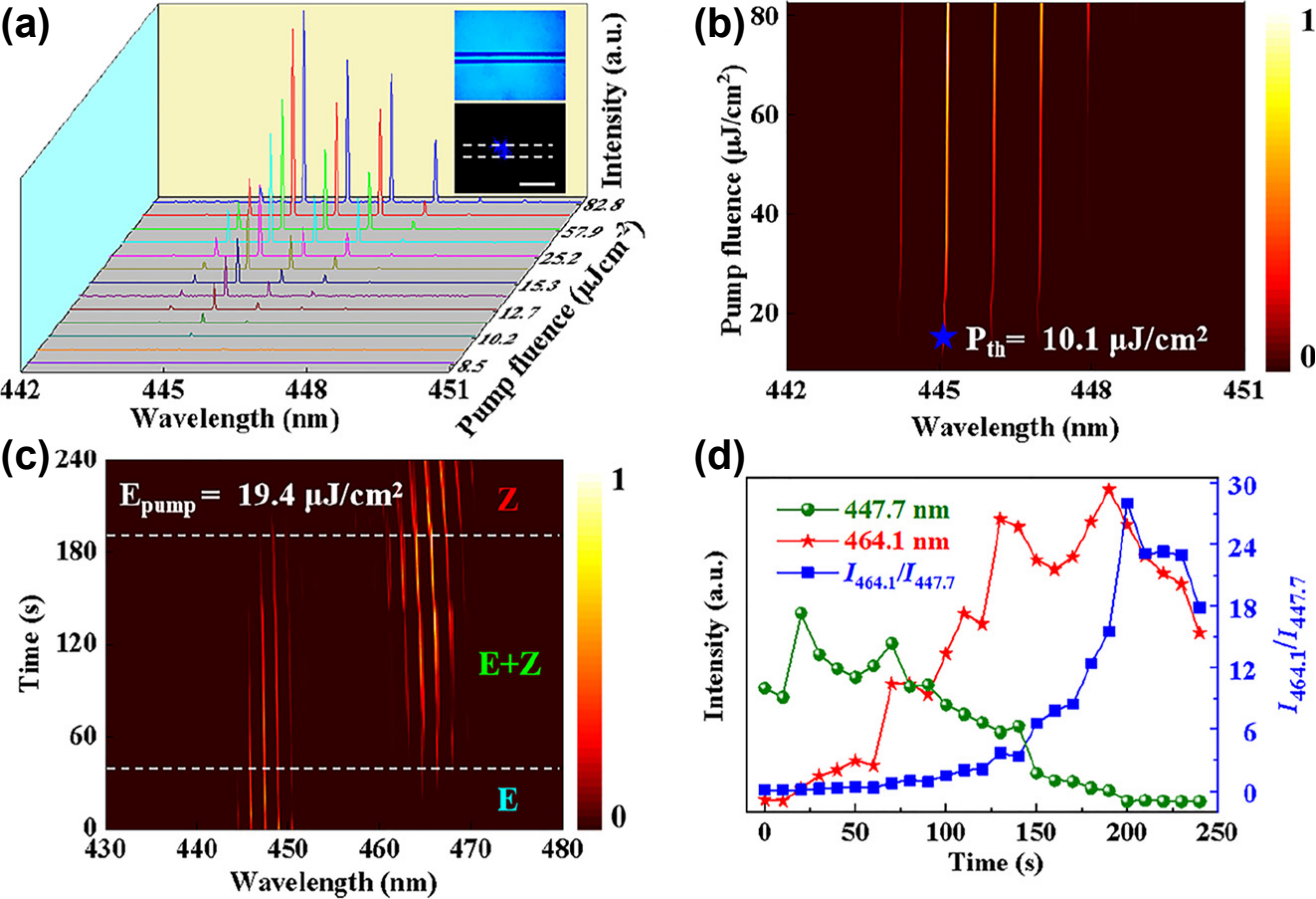
The two wavelength band emission WGM laser based on the ESIPT energy-level progress. (a) The lasing spectra for an individual microfiber under various different pump fluences. Inset: images of microfiber. Scale bar: 100 μm. (b) Relationship between pump fluences and WGM lasing intensity in an isolated S420@PVA microfiber. (c) The time-dependent multiwavelength switchable WGM lasing spectra with different ICT dye molecular excited state including *E*, *E* + *Z* and *Z* states. (d) Power-dependent profiles of lasing intensities around the mode peak 464.1 nm (red) and 447.7 nm (green), and the intensity ratio between two lasing modes (blue).

In experiment, the S420@PVA polymer microfiber with a doping concentration of 16 wt% is fabricated by a simple method. To protect the S420@PVA polymer microfiber and decrease the lasing threshold, the polydimethylsiloxane (PDMS) is applied as encapsulation agent on the surface of polymer compound microfiber. Firstly, a polymer microfiber with diameter of about 40 μm is selected to act as an excellent optical microcavity with smooth and uniform surface. It can be used as a high quality WGM microcavity to support optical feedback and oscillations. The S420@PVA microfiber exhibits a bright cylinder at the boundary (given in inset of Figure 2a) when pumped with a pulsed laser beam (*λ* = 343 nm, 1 ns). It predicts that a total internal reflection of the emitted light exists in the microfiber interface. In this situation, the WGM laser is anticipated on excitation of isolated polymer microfiber (S420@PVA) with a pulsed laser as displayed in Figure 2a, where different emission spectra were determined under various different pump fluences (i.e., from 8.5 μJ/cm^2^ to 82.8 μJ/cm^2^). It is seen that increase in pump fluence values led to respective increase in output density of PL emission spectra. Figure 2b demonstrates the relationship between pump fluences and WGM lasing output density in an isolated polymer microfiber. The WGM lasing of all mode has a full width at half maximum (FWHM) of less than 0.01 nm. The WGM lasing threshold (*λ* = 445 nm) for the blue-emitting is 10.1 μJ/cm^2^ and it is represented by star (Figure 2b).

Next, in order to investigate photo-isomerization effect on our proposed WGM laser, an isolated S420@PVA polymer microfiber with a diameter of 28 μm was fabricated. The pump fluence of about 19.4 μJ/cm^2^ was supplied. The infrared heating lamp is used as irradiation equipment. The ICT dye molecules can be transferred from *E* state to *Z* state via photo-isomerization. In the experiment, we collected data every 10 s. The time-dependent emission spectra of two wavelength band emission WGM cavity in different molecular excited states such as *E*, *E* + *Z,* and *Z* states is presented in Figure 2c. It is worth noting that the S420@PVA polymer-based microfiber cavity showed two emission regions: one emission band is at about 447.7 nm while other is at about 464.1 nm. When the excited state exists in *E*-state, only single band WGM wavelength peaks were appeared centered around 447.7 nm. Under photo-isomerization process, the *E* state of ICT dye molecules is transformed to *E* + *Z* state and finally to *Z* state inside the microcavity. The new lasing peaks were red shifted with center peak lies around 464.1 nm (Figure S2). Importantly, the two emission bands exhibits negative correlation. Further, it is noticed that the short wavelengths disappear and long wavelengths exist with irradiating. The intensity ratio (*I*_446.1_/*I*_447.7_) of the two emission wavelengths was calculated and illustrated in Figure 2d. In fact, this further testifies the existence of the two excited states (*E* state and *Z* state) under irradiation excitation.

The stability criteria in WGM laser is an important factor for various integrated photonic device applications. For stability analysis of our device, we recorded the lasing spectra at room temperature (Figure S3). Apart from this, the mode spacing can be tuned by changing the diameters of polymer microfiber as shown in Figure S4. Five number of modes were generated in WGM laser with a high-quality factor (i.e., *Q* = *λ*/Δλ) [38], [39], [40] of 12,500 for a peak found at 445 nm as shown in Figure S5. Importantly, the lasing mode number can be predicted by using the WGM equation (*mλ*_*m*_ = *πn*_eff_ *D*) [41] as shown in Figure S6. It is apparent from determined results that the S420@PVA polymer microfiber would be an excellent optical microcavity for lasing applications.

The electric field intensity distribution in transverse cross-section in the coupled microfiber cavity is simulated using commercial software COMSOL multi-physics 5.4 (Figure S7). Strong mode confinement is excited in the coupled system leading to the single mode lasing decided based on the mode selection mechanism^37^. The scanning electron microscope (SEM) image (Figure S8) displays that the coupled microfiber has excellent optical characteristics. The polymer microfiber can act as WGM cavity to support multiple modes resonant. The individual microfiber supported multiple modes on excitation with pump (Figure S9, top). However, when two microfibers of same material and dimensions were combined together leading to a single mode lasing as result of coupling upon excitation with pump source (Figure S9, bottom). The insets show the images of individual microfiber and the coupled microcavity.

**Figure 3:**
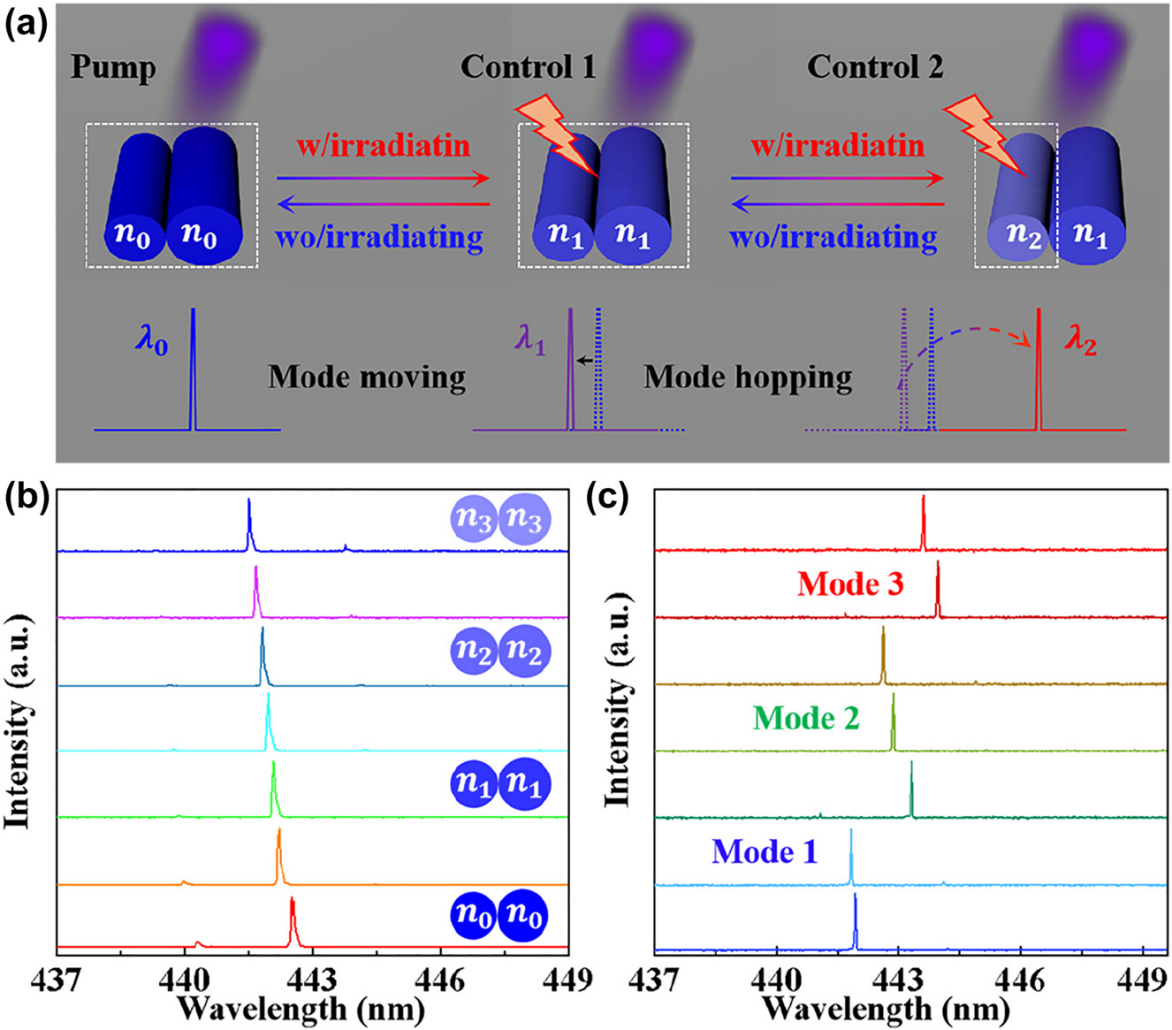
The switchable single mode lasing in microfiber coupled cavity. (a) Schematic illustration of the switchable single mode lasing in coupled microfiber. (b) Mode moving in the coupled microfibers activated by Control 1. Inset: illustration images of the coupled microfibers. (c) Mode hopping activated by Control 2 based on ESIPT progress.

Next, the coupled microfiber cavities were constructed in view of investigating the mode tunable and modulation impact on proposed WGM laser. Upon optical excitation, the switchable single mode lasing can be achieved based on the Vernier effect [42, 43]. As displayed in Figure 3a, the mode moving and mode hopping are achieved attributed to changing the effective refractive index and ESIPT progress, respectively. The single mode laser was blue shifted due to the decrease in effective refractive index of coupled microcavity as shown in Figure 3b, here the coupled microfibers are uniformly irradiated (Control 1). However, when only single microfiber from coupled microcavity was exposed to irradiance (Control 2), the mode hopping was observed from *λ*_1_ to *λ*_2_ owing to ESIPT progress [44].

**Figure 4:**
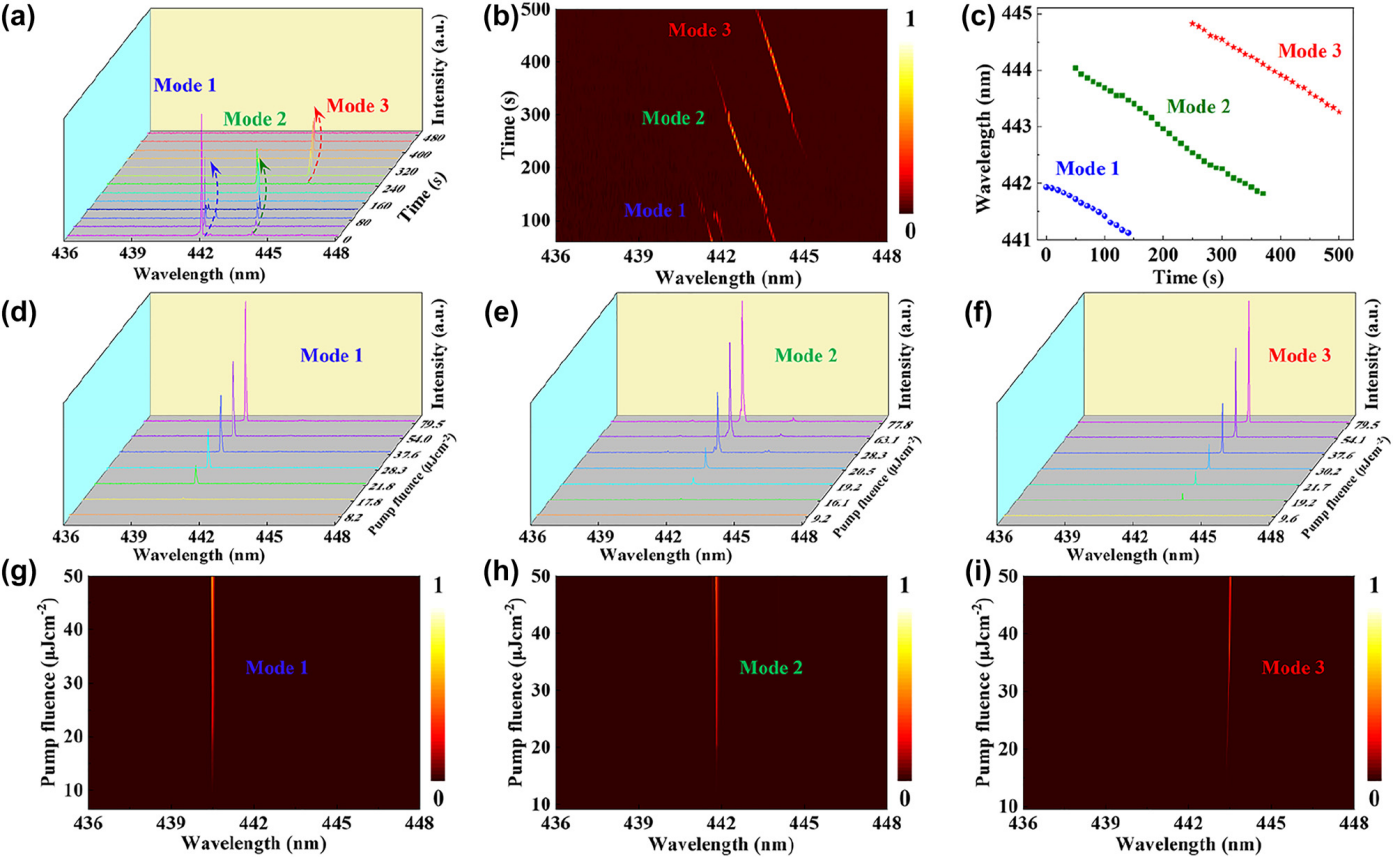
Mode selection in the coupled microfiber. (a) The temporal evolution of the lasing spectra in a microfiber coupled cavity. (b) Mapping of the lasing profile of the single mode lasing from mode 1, mode 2 to mode 3. (c) Time-dependent laser mode in the coupled microfibers. (d–f) PL spectra with mode 1, 2, 3 with different pump fluences in coupled microfibers. (g–i) Relationship between pump fluences and output intensity in the coupled microcavity.

Figure 4(a)–(c) displays the single mode lasing shift in the coupled microfibers with passage of time. The laser mode in the coupled cavity system was red shifted and termed as mode 1, mode 2, and mode 3 upon irradiance by infrared heating lamp (Figure S10). As displayed in Figure 4(d)–(f), the output density of three lasing modes was increased with variation in pump fluence values. Each single mode exhibited FWHM with value less than 0.01 nm and the lasing threshold was determined around 20 μJ/cm^2^ as shown in Figure 4(g)–(i). In view of these, the two wavelength band emission WGM laser with low-threshold can be achieved based on a typical ESIPT process.

**Figure 5:**
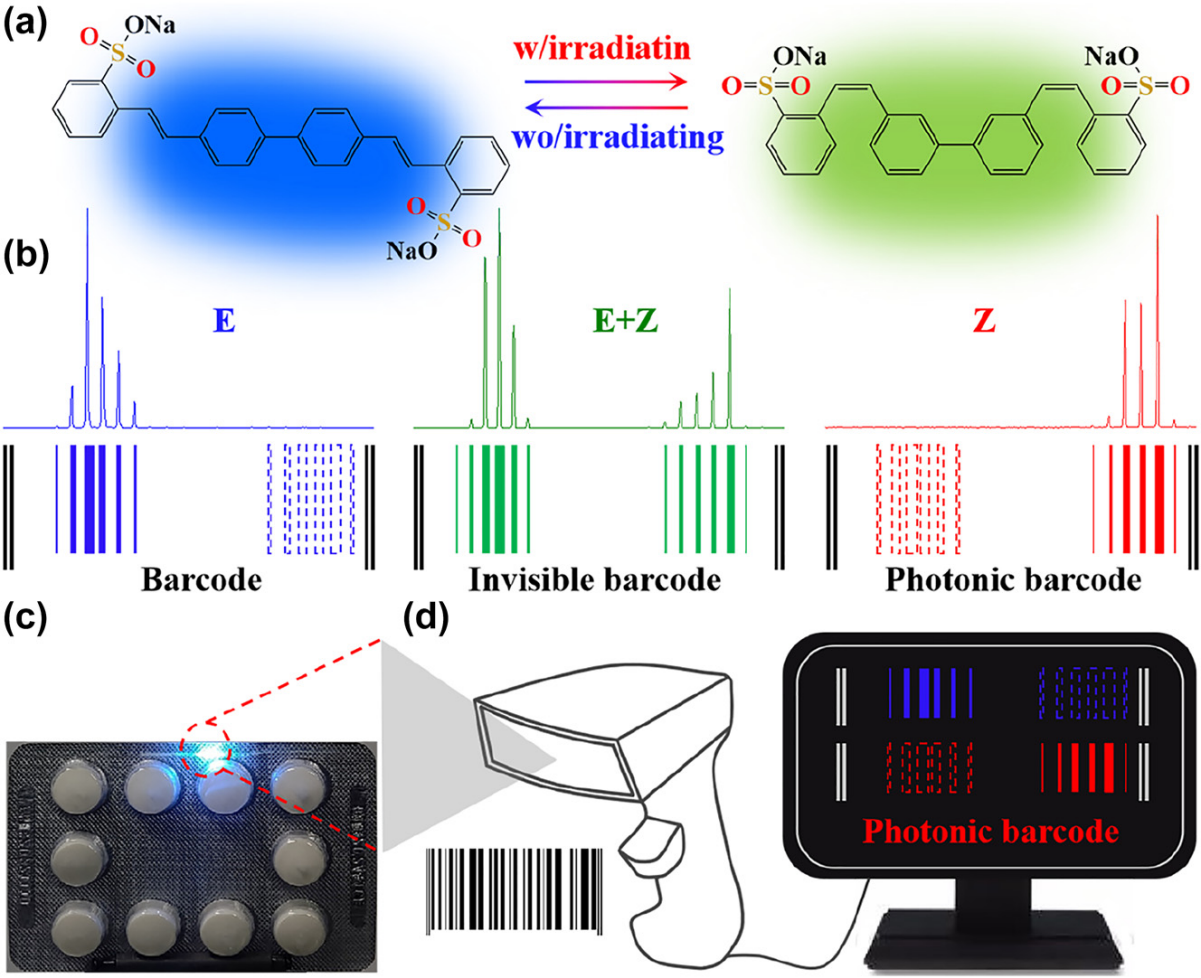
Design and fabrication of the photonic barcodes. (a) Highly polarized organic ICT dye molecular structures with trans-S420 state and cis-S420 state, respectively. (b) The PL spectra and photonic barcodes from a typical microfiber. (c–d) Proof-of-concept emonstration of a microfiber based on photonic barcodes for anti-counterfeiting.

Based on the external stimuli responsiveness, smart responsive materials was developed for the fabrication of photonic barcodes generation which may find applications in information security and anti-counterfeiting [45]. Here, highly polarized organic ICT dye molecular is chosen as the responsive acceptor to achieve photonic barcodes due to its characteristics of photo-isomerization as displayed in Figure 5a. As mentioned earlier, S420 dye molecular structure has two states with trans-S420 state and cis-S420 state. Upon excitation by a pulsed pump laser, the PL spectrum of single optical fiber was obtained and given in Figure 5b. Here, the designed barcodes can be generated by following the strategy as: the solid bar corresponds to mode wavelength with bar width is set proportion to PL intensity of respective selected mode (in Figure 5b). Following this scheme, one new photonic barcode is generated for same cavity after exposure to irradiance, which is closely related to the original barcode with slight variation (Figure S11). The new photonic barcode is used as “security tag”, considering the smart responsiveness activated by photo-isomerization. As a proof-of-concept demonstration, the microfiber is attached to a drug package as the “security tag” as displayed in Figure 5(c) and (d). The photonic barcode can further be extended to other secret media, such as identity documents, passport and food.

## 4 Conclusions

In summary, the two wavelength band emission WGM lasers are achieved in polymer microfibers doped with highly polarized organic ICT dye molecules. The photo-isomerization activated and modulated ICT process can result in two optical gain regions. Meanwhile, the mode moving is induced by changing the effective refractive index. Based on the photo-isomerization activated, we achieved switchable single mode lasing in coupled microfiber cavity. Our results offer guidance for the development of two wavelength band emission WGM laser towards photonic integration applications.

## Supplementary Material

Supplementary Material Details
