# Supplementary material for: Two wavelength band emission WGM lasers via photo-isomerization
Source: Nanophotonics. 2023 Oct 20;12(21):4087–94. doi: 10.1515/nanoph-2023-0522 (PMC11502004; doi:10.1515/nanoph-2023-0522)
Supplement: Supplementary file 1 — Supplementary Material Details [file j_nanoph-2023-0522_suppl_001.docx]

**Supporting Information**

**Two wavelength band emission WGM lasers via photo-isomerization**

Kun Ge, Jun Ruan, Ningning Liang, Dan Guo, Libin Cui, Naeem Iqbal, Tianrui Zhai*

Faculty of Science, Beijing University of Technology, Beijing, 100124, China.

*E-mail: [trzhai@bjut.edu.cn](mailto:trzhai@bjut.edu.cn)

**Table of Contents**

**[1. The fluorescence spectra in different polar solvents 2](#_Toc132135034)**

**[2. Switchable WGM laser with photo-isomerization 3](#_Toc132135035)**

**[3. The stability of WGM laser in isolated microfiber 5](#_Toc132135036)**

**[4. The relationship between diameter and FSR 6](#_Toc132135037)**

**[5. The](#_Toc132135038) *[Q](#_Toc132135038)* [factor of WGM laser 7](#_Toc132135038)**

**[6. The relationship between mode number and peak wavelength 8](#_Toc132135039)**

**[7. Numerical simulation of electric field distribution 9](#_Toc132135040)**

**[8. The SEM images with microfiber coupled cavity. 10](#_Toc132135041)**

**[9. The lasing spectra in isolated microfiber and coupled microcavity 11](#_Toc132135042)**

**[10. Mode selection in fiber coupled microcavity 12](#_Toc132135043)**

**[11. The spectral distribution of solar radiation 13](#_Toc132135043)**

**[12. References 14](#_Toc132135044)**

## **The fluorescence spectra in different polarity solvents**

For the highly polarized intramolecular charge-transfer (ICT) dye molecule, the emission peak will be red-shifting with increasing solvent polarity [1]. To investigation the polar solvents influence on fluorescence spectra of highly polarized organic ICT dye molecule, we produce ICT dye solutions in different solvents with concentration of 10 mg/ml. In our experiment, we act S420 dye as ICT dye material. The fluorescence spectra of S420 dye molecule with different polarity solvents are as shown in Figure S1. In acetone, the PL emission peak of S420 is at 427.5 nm. With the increase of solvent polarity, the PL emission peak shifts to 437.9 nm in methanol solution, 454.2 nm in dimethyl sulfoxide (DMSO) solution and 457.1 nm in maxed solution with DMSO and water . Finally, the PL emission peak of S420 undergoes a large red shifted of 43 nm in water (470.6 nm) versus in acetone (427.5 nm). This solvatochromic shift is duo to the strong ICT character of S420 dye molecules [2].


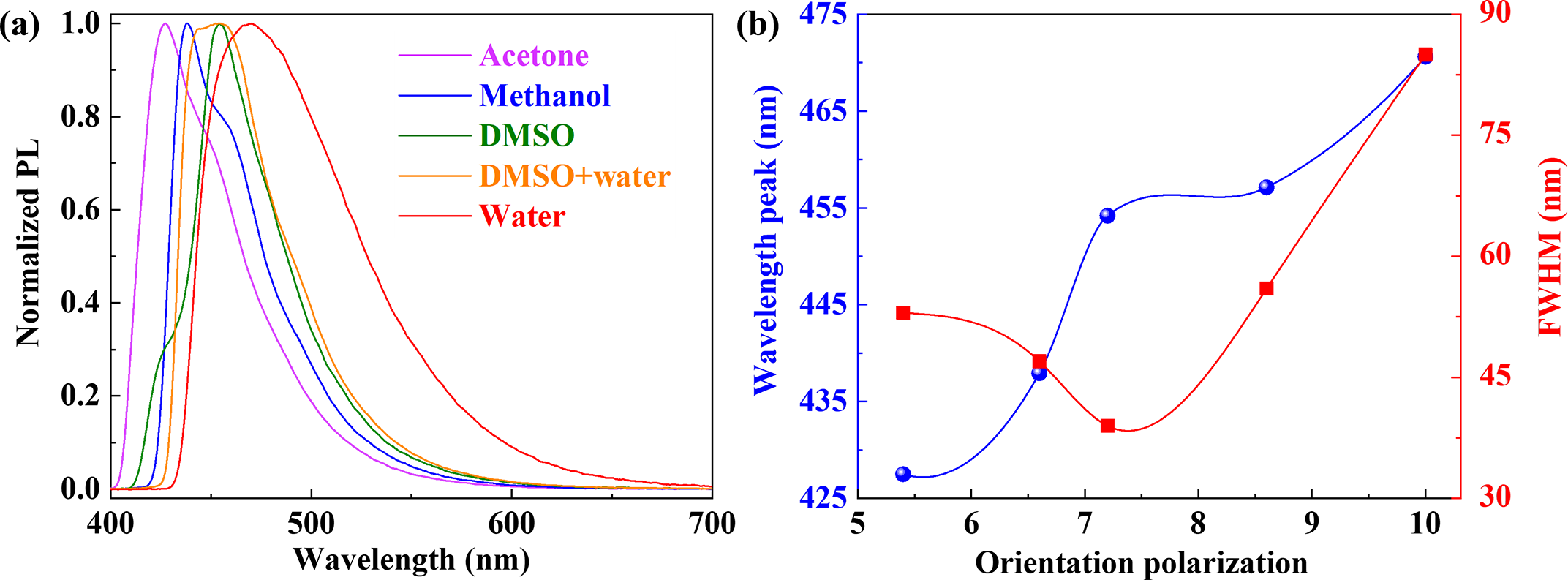


Figure S1. The fluorescence spectra of ICT dye molecule with different polarity solvents. (a) Normalized PL spectra of S420 dye molecule in different solvents with increasing polarities. (b) The relationship between wavelength peak/FWHM and orientation polarization of the solvents.

1. **Switchable WGM laser with photo-isomerization**

In our experiment, an organic polymer microfiber of about 28 μm is selected to act as an excellent optical microcavity. The switchable WGM laser with photo-isomerization based on excited-state intramolecular proton transfer (ESIPT) process is achieved when the polymer microfiber is pumped by pulsed laser beam as shown in Figure S2a. The polymer microfiber cavity shows two emission regions: one emission band is at about 447.7 nm and another emission band is at about 464.1 nm. Lasing emission peak is about 447.7 nm with trans-form (E) structure. The new lasing peak of 464.1 nm appears when emission regions excite state is from E structure to cis-form (Z) structure [3-4]. Under photo-isomerization activated, the emission peak is from 447.7 nm to 464.1 nm based on the ESIPT process as shown in Figure S2b. We keep the pump density stable with 19.4 μJ/cm^2^ in the experiment. The WGM laser intensity increase with the center peak lies about 464.1 nm (long wavelength) with the ICT dye molecular excited stated from E state to Z state. However, the gain medium is affected by photobleaching, resulting a decrease in photoluminescence efficiency. The intensity of WGM lasing spectra will decrease.


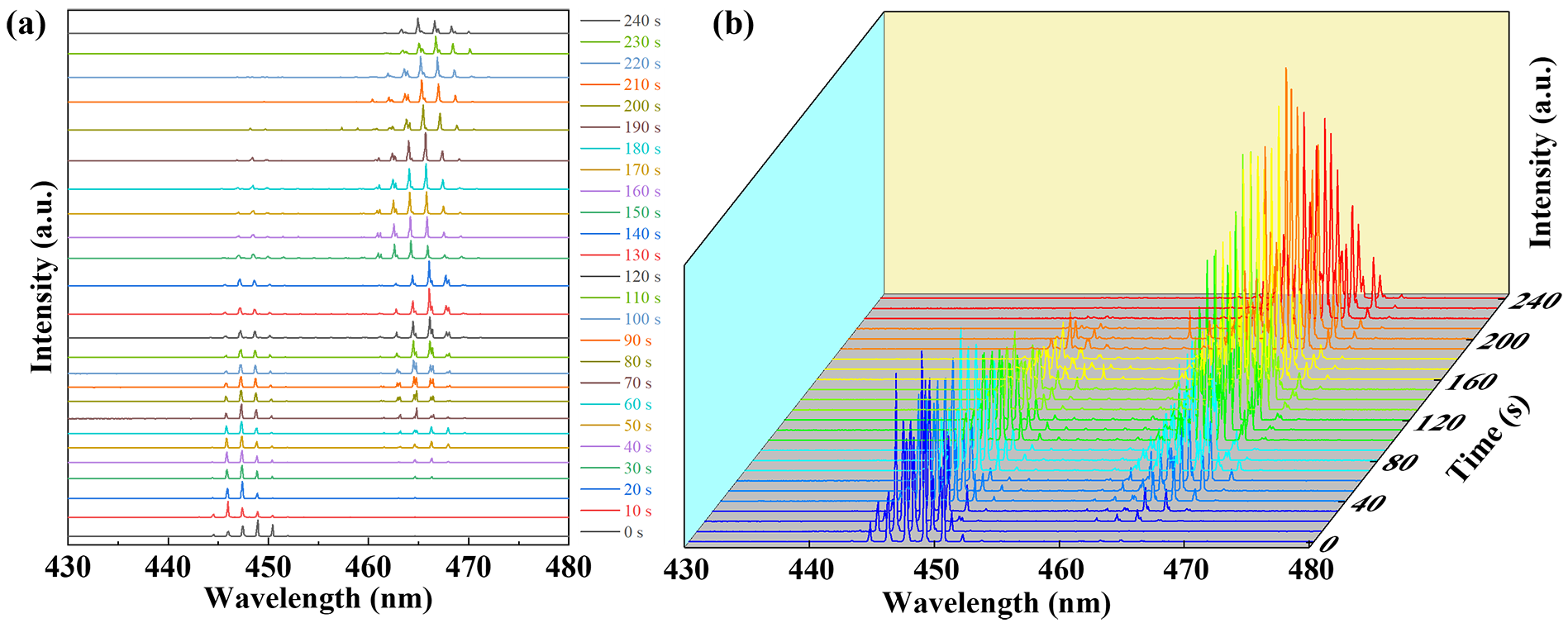


Figure S2. The switchable WGM laser with photo-isomerization activated based on ESIPT process in polymer microfiber cavity. (a) WGM laser spectra is obtained with diameter of 28 μm in isolated polymer microfiber. (b) The evolution of WGM laser is with different dye molecular excited staste.

Table 1. Nonexhaustive list of recent wavelength tunable lasers

| Microcavity structure | *Q* factor | Physical mechanism | Tunable range | Reference |
| --- | --- | --- | --- | --- |
| Microring | 1.76×10^4^ | Temperature | 0.92 nm | Wan *et al*, 2017^[5]^ |
| Polymer film | <3×10^3^ | Humidity | N.A. | Tong *et al*, 2017^[6]^ |
| Microfiber | 6×10^3^ | Temperature | <1 nm | Ge *et al*, 2021^[7]^ |
| Metal-organic frameworks | 3×10^3^ | Intramolecular  charge transfer | >40 nm | Wei *et al*, 2016^[8]^ |
| Microbubble | N.A. | Aerostatic  pressure | N.A. | Lu *et al*, 2016^[9]^ |
| Microfluidic channels | 8×10^3^ | Re-absorption | 16 nm | Niu *et al*, 2021^[10]^ |
| Nanowire | <5×10^3^ | Excited-state Intramolecular  charge transfer | 23 nm | Yan *et al*, 2015^[11]^ |
| Microfibers | 1.25×10^4^ | Excited-state Intramolecular  charge transfer | 16.4 nm | This work |

1. **The stability of WGM laser in isolated microfiber**

The stability of WGM laser is important to various integrated photonic devices. To investigate the stability of the WGM laser, the lasing spectra is recorded every 10 seconds at room temperature. Figure S3(a-c) shows the lasing spectra evolution of peak wavelength and emission intensity with different pump time in isolated polymer microfiber cavity. The peak wavelength is little changed with increasing the pump time as shown in Figure S3d. The results demonstrate that the polymer microfiber lasing has excellent optical characteristics.


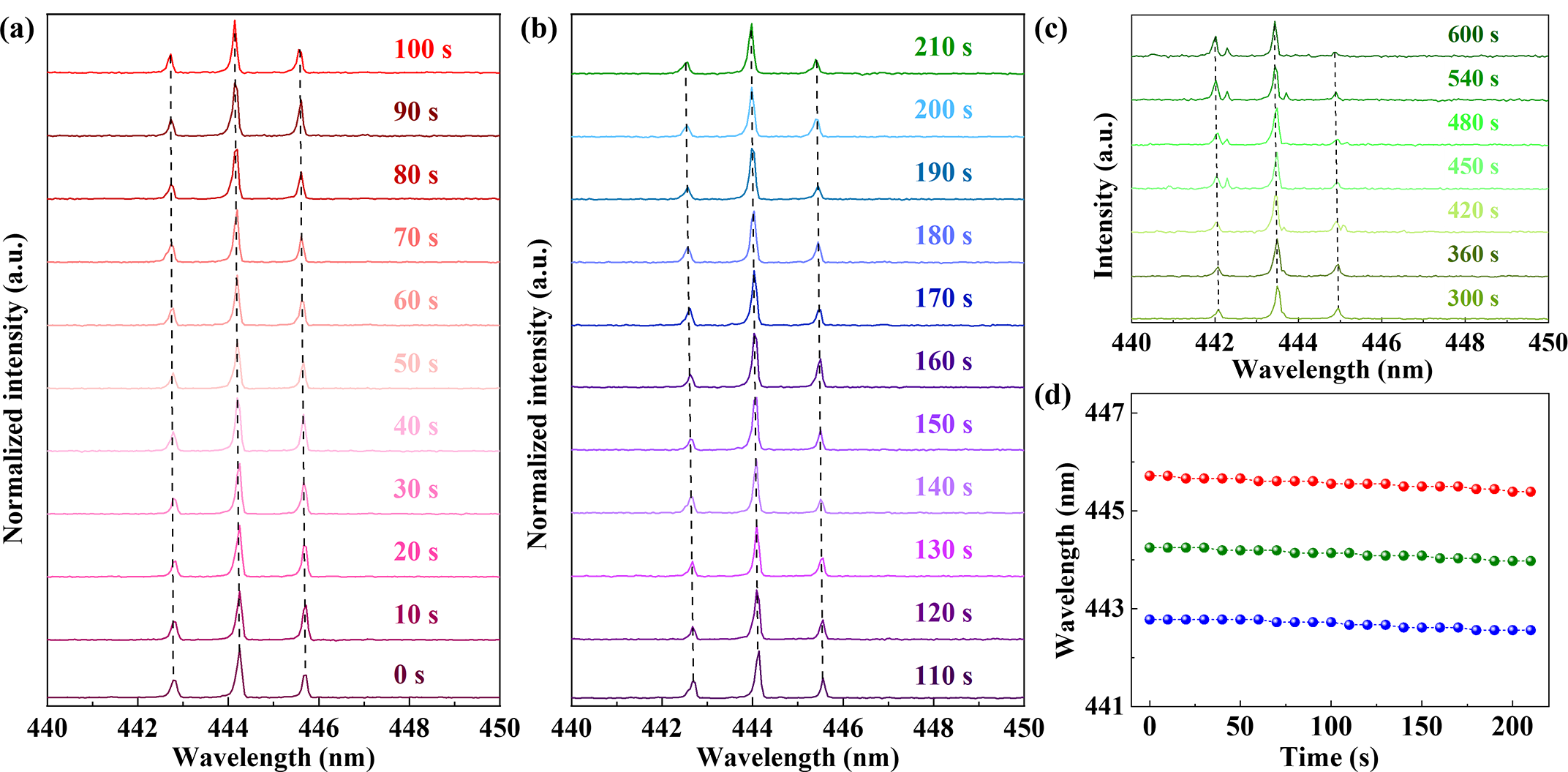


Figure S3. The stability of WGM laser in polymer microfiber.

1. **The relationship between diameter and FSR**

The mode spacing can be tuned by changing the diameters of polymer microfiber. The mode spacing is decreased with increase of the polymer microfiber cavity diameter as displayed in Figure S4a. The free spectrum range (FSR) values are calculated as 0.61 nm, 1.16 nm, 1.47 nm and 1.82 nm against the diameters of polymer microfibers 67 μm, 35 μm, 27 μm, and 22 μm, respectively. Figure S4b illustrates the relationship between diameter and FSR. The red solid curve is fitted by the WGM equation:

$\mathrm{FSR}=\frac{\lambda^{2}}{\pi Dn_{\mathrm{eff}}}$ (S1)

where *λ* represents the center wavelength, *n*_eff_ is the effective refractive index, and *D* is the diameters of polymer microfiber.


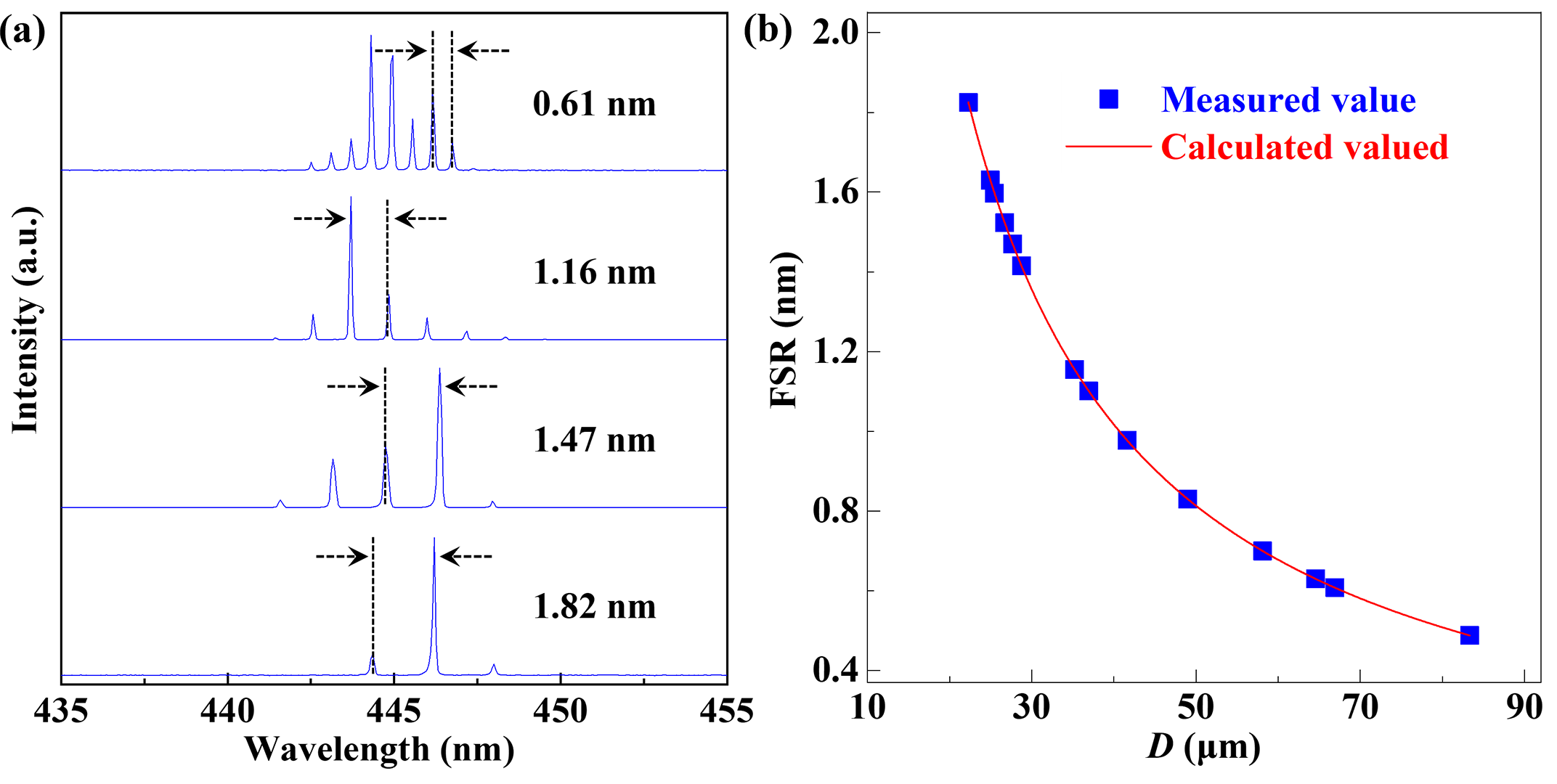


Figure S4. The relationship between diameter and FSR. (a) Measured emission spectra of WGM lasing with different diameters. (b) Relationship between FSR and *D*.

1. **The *Q* factor of WGM laser**

The *Q* factor of WGM laser is an important parameter in the optical microcavity and one of the important parameters used to measure the excellent performance of microcavities The *Q* factor can be determined as [12]:

$Q=\omega\frac{W}{-dW/dt}=\omega\tau=\frac{\lambda}{\lambda_{\mathrm{FWHM}}}$ (S2)

Where $\omega$ represents the resonant angular frequency of the resonant mode, *W* represents the total energy stored in the cavity, $-dW/dt$ indicates the dissipated energy in the cavity per unit time, $\tau$represents the photon lifetime of the microcavity light field, *λ* and *λ*_FWHM_ are the peak wavelength and corresponding FWHM, respectively. Figure R1a shows the multiple mode WGM laser with diameter of 45 μm. The calculation of *Q* value is about 12500 as displayed in Figure S4b. The high *Q* factor demonstrates that the microfiber cavity has low optical loss and high sensitivity. The single mode lasing in the coupled microfiber cavity is as shown in Figure R1c, and the *Q* factor is about 6500 as displayed in Figure S4d.

The *Q* value of the microcavity is negatively correlated with the energy dissipation within the cavity, and the total intrinsic loss consists of three parts: radiation loss *Q*_rad_, absorption loss *Q*_abs_, and scattering loss *Q*_sca_. Then use the formula to express:

$\frac{1}{Q_{\mathrm{int}}}=\frac{1}{Q_{\mathrm{rad}}}+\frac{1}{Q_{\mathrm{abs}}}+\frac{1}{Q_{\mathrm{sca}}}$ (S3)

The *Q* factor will decrease when two microfibers from the coupling system.


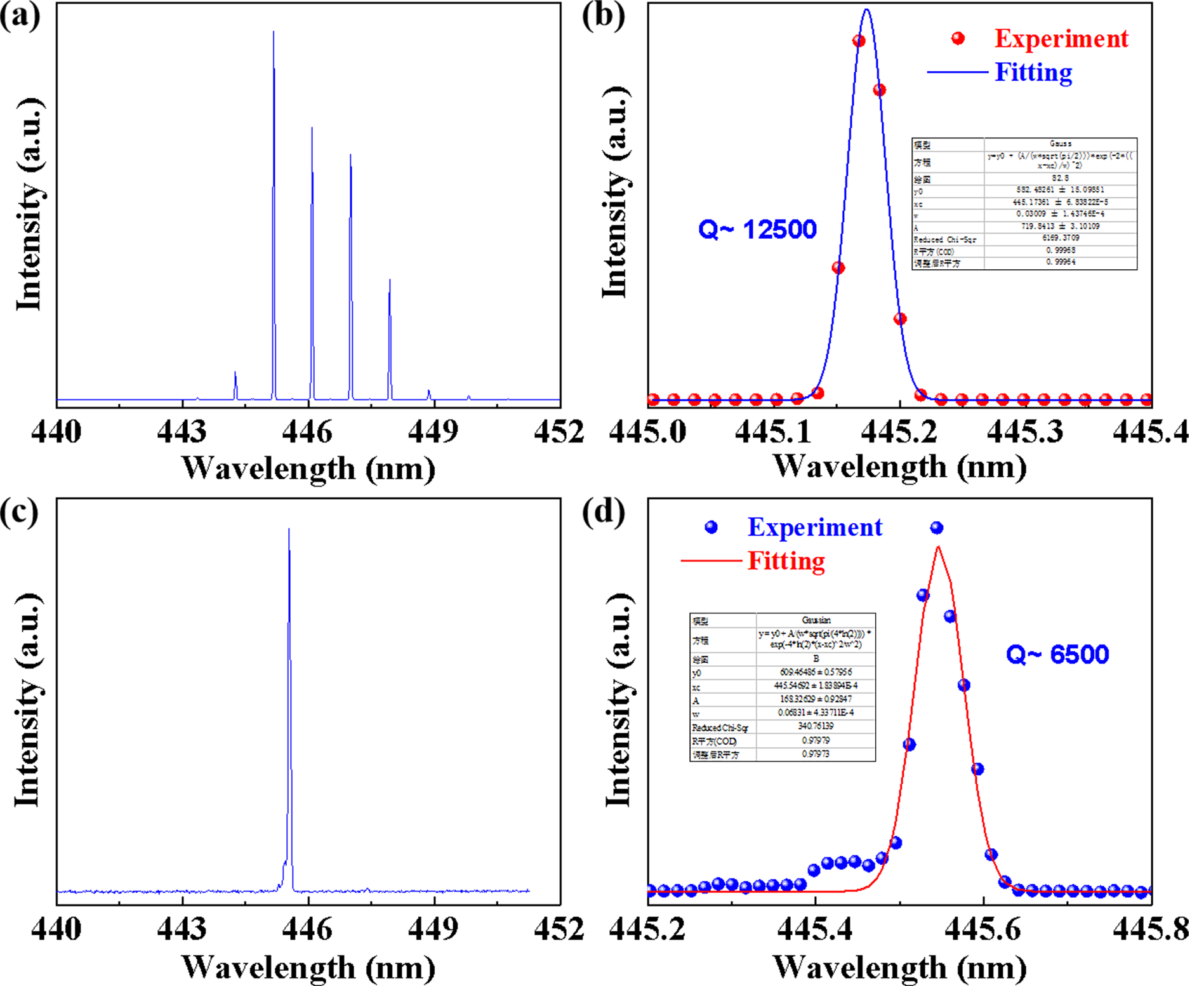


Figure S4. The WGM lasing and *Q* factor. (a) The multiple mode WGM laser spectra in individual microfiber cavity. (b) The *Q* factor (~12500) of resonant mode with wavelength 455.15 nm. (c) The single mode lasing in the coupled microfiber cavity. (d) The *Q* factor (~6500) of resonant mode.

1. **The relationship between mode number and peak wavelength**

The spectral map of the normalized emission intensity is shown in Figure S6. The mode number can be predicted by the WGM equation (*mλ*_m_=π*n*_eff_*D*), here *m* is mode number, *λ* is respective resonant wavelength, *D* is diameter of microfiber and *n*_eff_ is effective refractive index of microfiber under investigation [13].


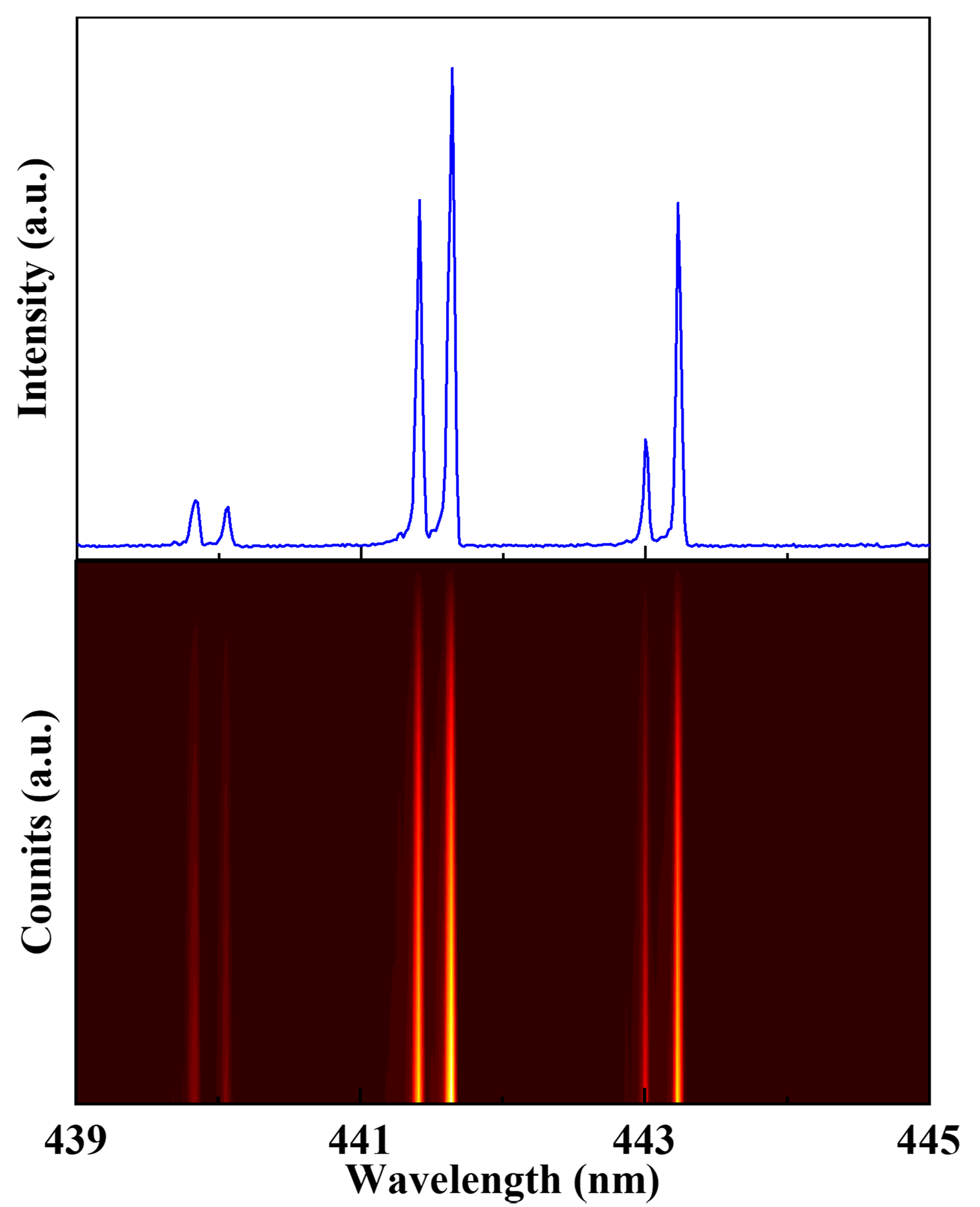


Figure S6. The normalized emission spectra (top) and spectral map of the emission intensity (bottom)

1. **Numerical simulation of electric field distribution**

There are two type coupling modes in the coupled microcavity: bonding mode and antibonding mode. Figure S7 demonstrates the numerically simulated electric field distributions of the lasing modes, which reveals the mode modulation in the coupled microcavity. The energy of the photons can be well confined in the microfibers, and the radial scattering into the air is quite limited. The single mode lasing will be excited when the same resonant mode exists in the two isolated organic polymer microfibers. And other resonant mode will be suppressed.


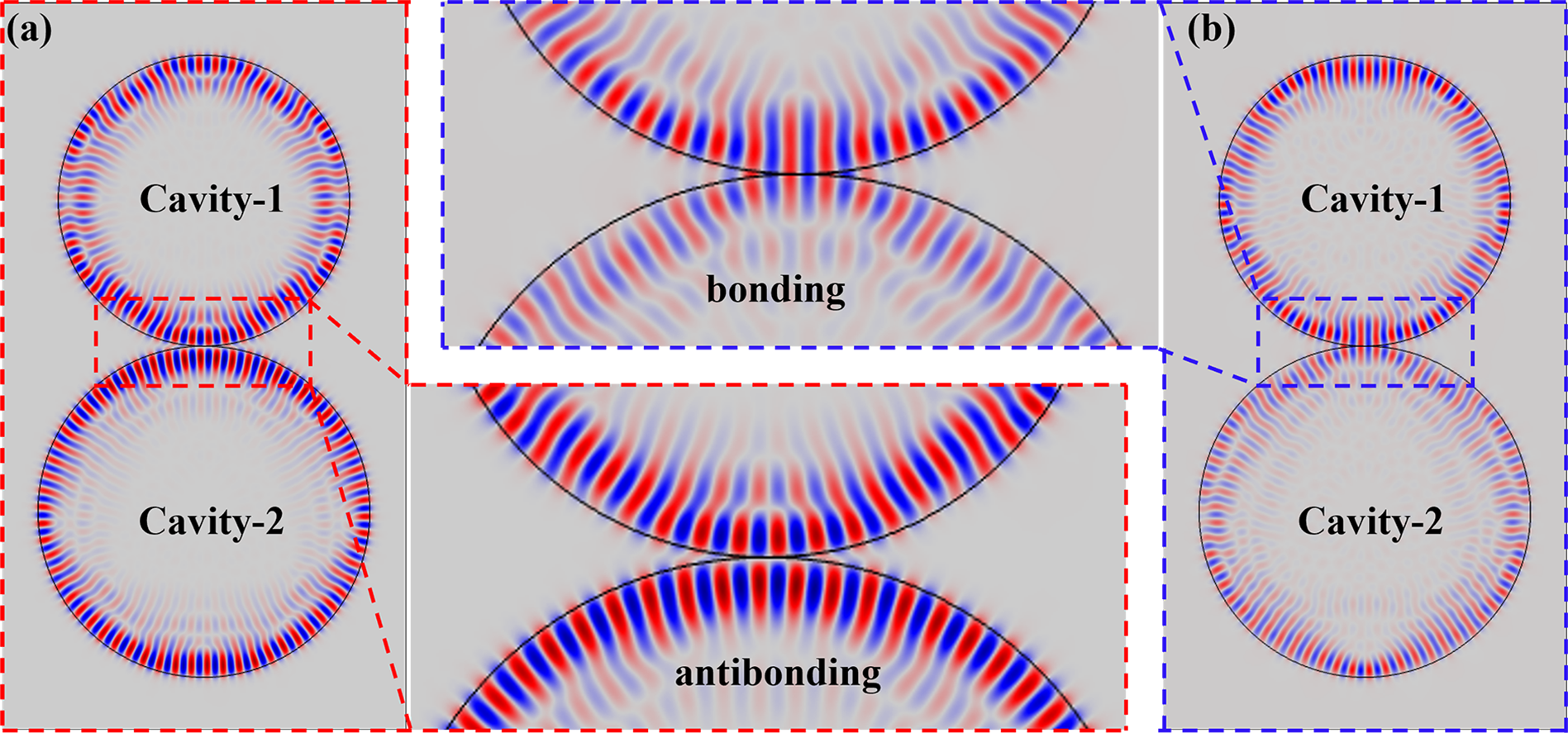


Figure S7. Numerical simulation of electric field distribution in coupled microfiber.

1. **The SEM image with microfiber coupled cavity**

In order to gain more details about the surface of polymer microfiber coupled cavity, the predicted SEM image of coupled microfiber cavity is as shown in Figure S8. The results demonstrate that polymer microfiber has excellent optical property.


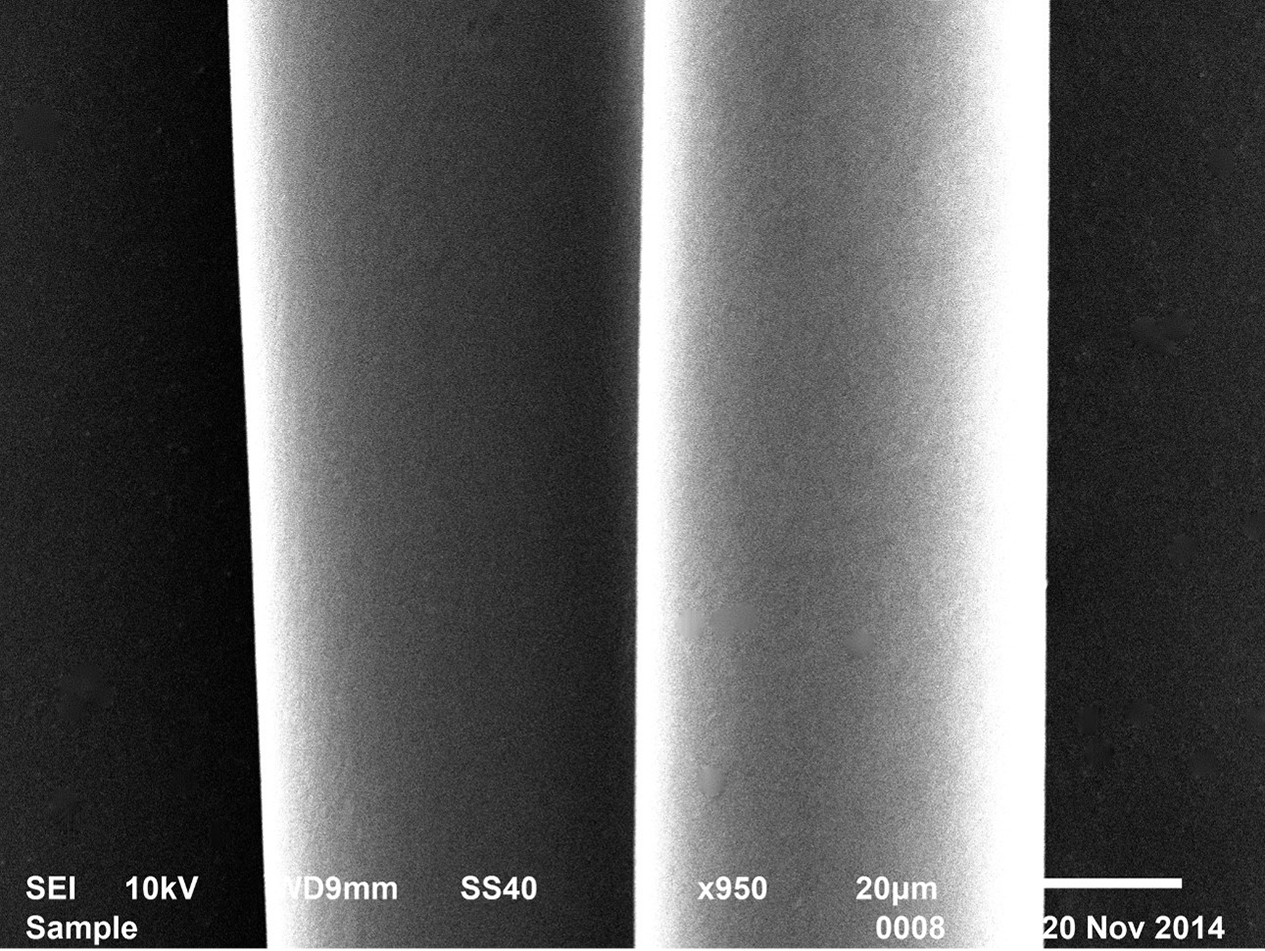


Figure S8. The SEM images with microfiber coupled cavity. Scale bar: 20 μm.

1. **The lasing spectra in isolated microfiber and coupled microcavity**

The polymer microfiber has excellent optical property, which can act as WGM cavity to support multiple mode resonant. The WGM lasing can be achieved when the polymer microfiber is pumped in the isolated microcavity as shown in Figure S9 (top). One of the lasing modes from microfiber cavity is selected when another microfiber is coupled with the cavity, leading to the blue single mode lasing (in Figure S9, bottom). The insets show the images of the individual microfiber and the coupled microfibers.


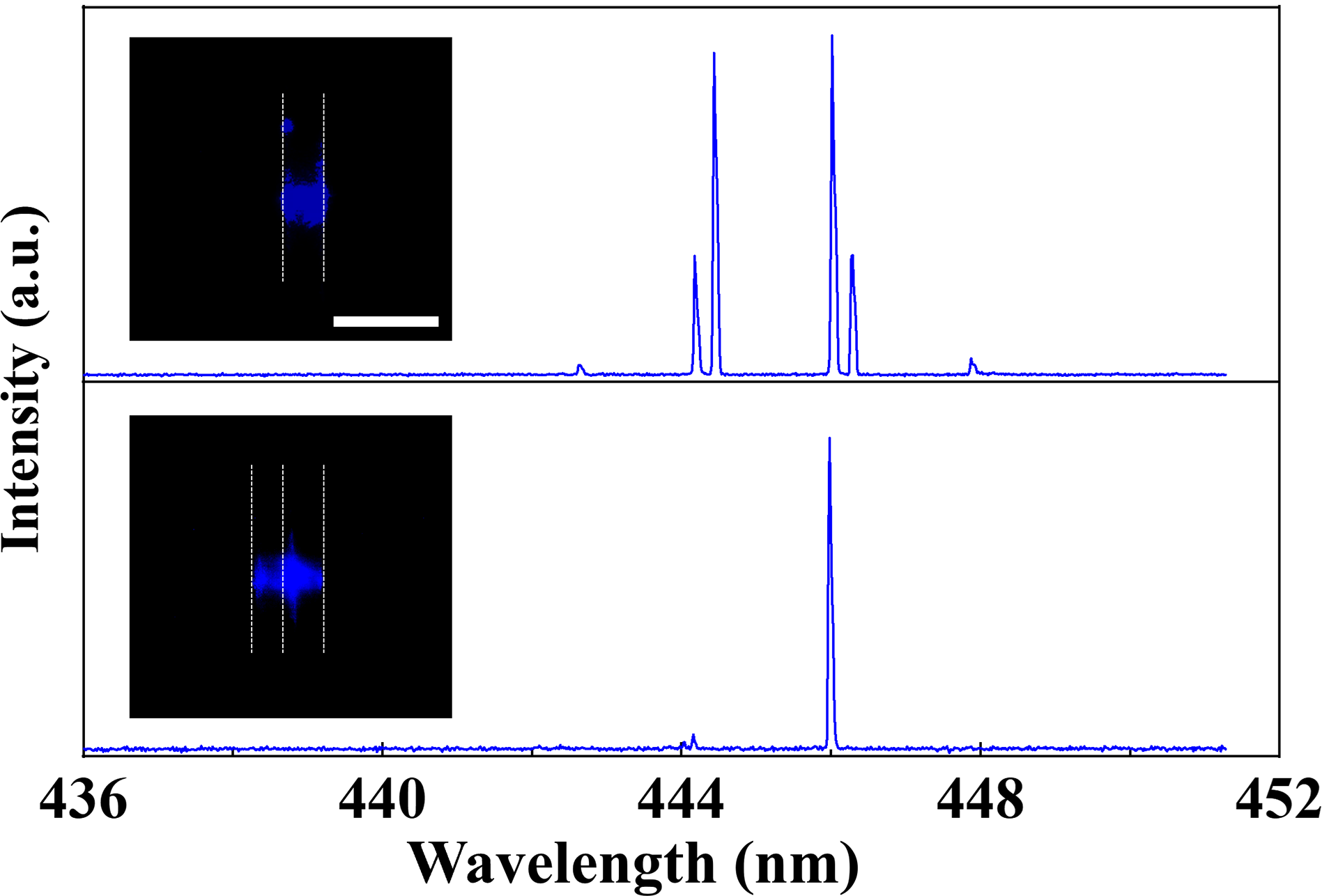


Figure S9. The lasing spectra in isolated microfiber and coupled microcavity. Inset: The PL images of the isolated microfibers (top) and coupled microcavity (bottom). Scale bar: 100 μm.

1. **Mode selection in fiber coupled microcavity**

Coupled microfiber cavities doped with S420 dye molecule are constructed to achieve the single mode lasing duo to Vernier effect. Once the coupled microfibers are exposed to uniformly irradiating, the single mode lasing will move towards the shorter wavelength duo to the decrease in effective refractive index. When only one polymer microfiber is irradiated, the mode match and selection will occur at another position, thereby leading to laser mode hopping based on ESIPT energy-level progress [14]. The thermal-induced resonant wavelength shift can be expressed as follows: [15-16]

$d\lambda=\lambda\left( \frac{1}{n_{\mathrm{eff}}}\frac{dn_{\mathrm{eff}}}{dT}+\frac{1}{D}\frac{dD}{dT} \right)dT$ (1)

where *n*_eff_ is the effective refractive index of the polymer microfiber, *D* is the diameter of the microcavity, d*n*_eff_/d*T* and $\frac{1}{D}\frac{dD}{dT}$ denote the thermal-optic effect and thermal expansion effect of the active materials and surrounding environment. The diameter of the solid microcavity is insensitive to the temperature. The thermal expansion coefficient of the solid material is small (10^−6^/°C) [17]. In the experiment, we can ignore the effect of thermal expansion coefficient and only consider the influence of thermal-optic coefficient. Moreover, the effective refractive index of gain materials decreases with increasing the temperature. In other words, the thermo-optic coefficient (d*n*_eff_/d*T*) of the active material is negative. Therefore, the results can be predicted theoretically according to the WGM equation (m*λ*=π*nD*), which the peak wavelength is also blue-shifting with increasing the temperature. The change in effective refractive index is physical change that does not alter the properties of the material. Therefore, the change in refractive index is reversible without irradiation.


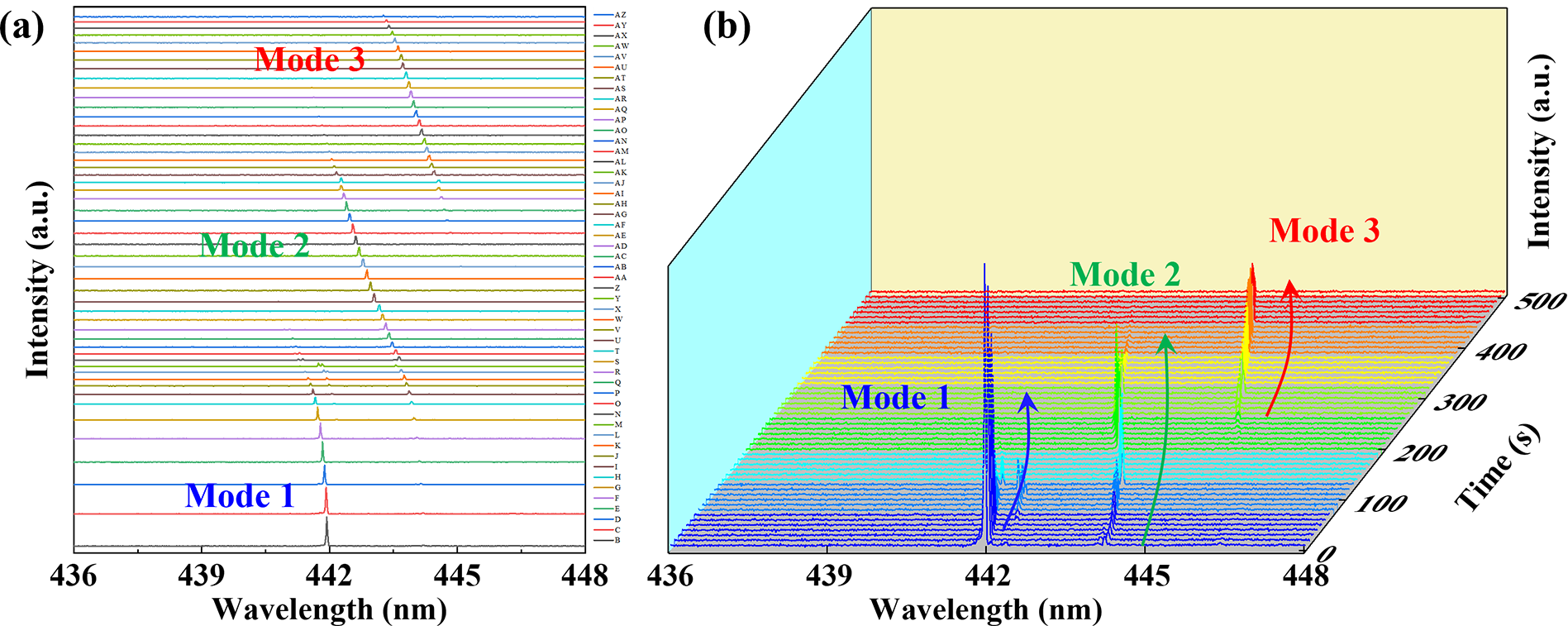


Figure S10. Switchable single mode lasing in the coupled microfibers.

1. **The spectral distribution of solar radiation**

The barcodes will change under the illumination of sunlight. The infrared heating lamp is used as irradiation equipment in our experiment, which has the characteristics of energy focusing and high power density. However, the absorption efficiency of WGM microcavity for sunlight is lower than that of irradiating.


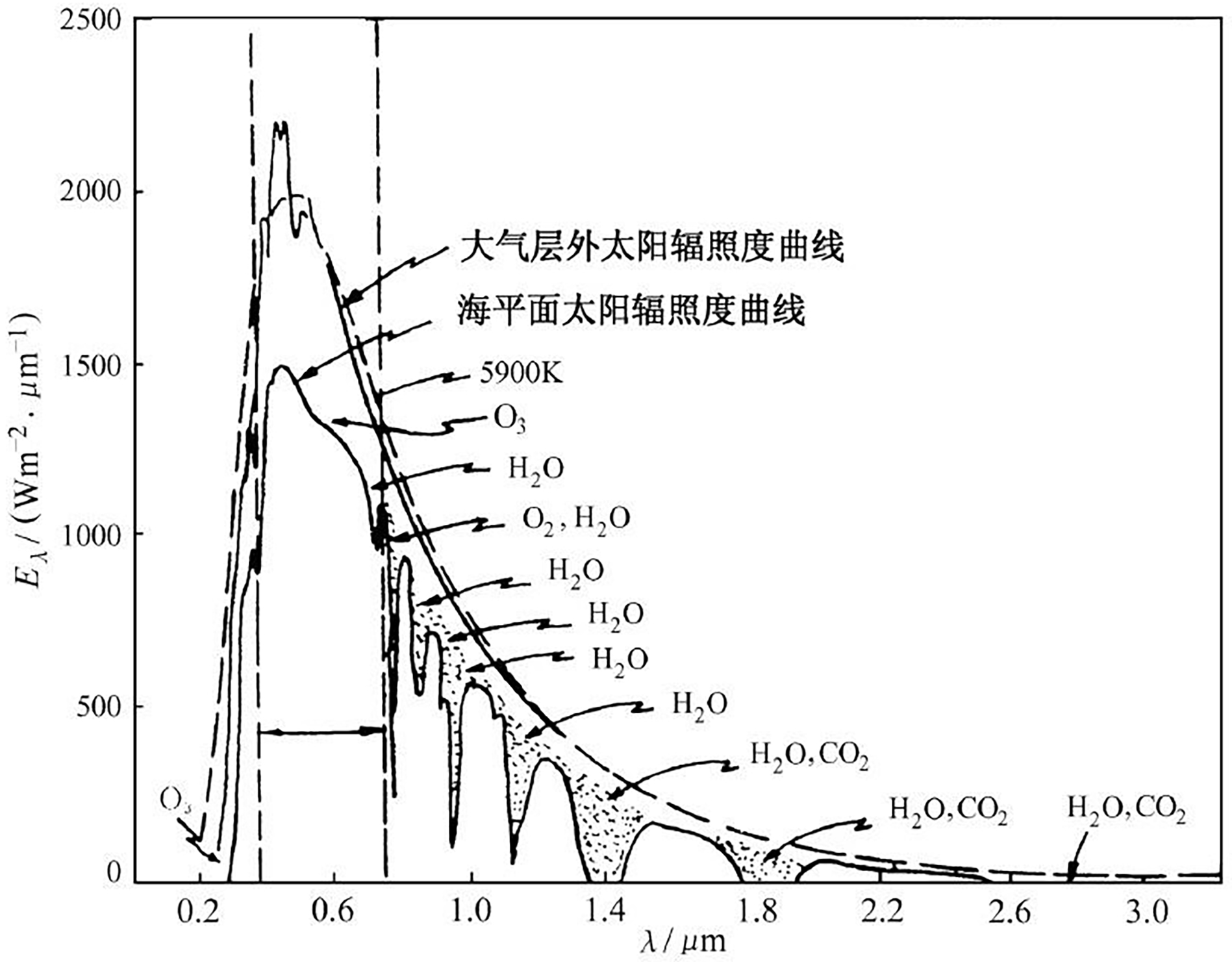


Figure S11. The spectral distribution of solar radiation (From the network).

1. **References**
2. W. Li, D. Liu, F. Shen, et al. “A twsting donor-acceptor molecule with an intercrossed excited state for highly efficient, deep-blue electroluminescence,” *Adv. Funct. Mater.*, vol. 22, pp. 2797, 2012.
3. H. Dong, Y. Wei, W. Zhang, et al. “Broadband tunable microlasers based on controlled intramolecular charge-transfer process in organic supramolecular microcrystals,” *J. Am. Chem. Soc.*, vol. 138, pp. 1118-1121, 2016.
4. Q. Huang, Q. Guo, J. Lan, J. You, “Tuning the dual emission of keto/enol forms of excited-state intramolecular proton transfer (ESIPT) emitters via intramolecular charge transfer (ICT),” *Dyes Pigments*, vol. 193, pp. 109497, 2021.
5. M. Berggren, A. Dodabalapur, R. Slusher, Z. Bao, “Light amplification in organic thin films using cascade energy transfer,” *Nature*, vol. 389, pp. 466-469, 1997.
6. L. Wan, H. Chandrahalim, C. Chen, et al. “[On-chip, high-sensitivity temperature sensors based on dye-doped solid-state polymer microring lasers](https://www.x-mol.com/paperRedirect/336842),” *Appl. Phys. Lett.*, vol. 111, pp. 061109, 2017.
7. J. Tong, X. Shi, Y. Wang, L. Han, T. Zhai, “Flexible plasmonic random laser for wearable humidity sensing,” *Sci. China Inf. Sci.*, vol. 64, pp. 222401, 2021.
8. K. Ge, D. Guo, B. Niu, Z. Xu, J. Ruan, T. Zhai, “Pump-controlled RGB single-mode polymer lasers based on a hybrid 2D-3D μ-cavity for temperature sensing,” *Nanophotonics,* [vol. 10, p](https://www.degruyter.com/journal/key/nanoph/10/18/html)p, 4591-4599, 2021.
9. Y. Wei, H. Dong, C. Wei, W. Zhang, Y. Yan, Y. Zhao, “Wavelength-tunable microlasers based on the encapsulation of organic dye in metal-organic frameworks,” *Adv. Mater.*, vol. 28, pp. 7424-7429, 2016.
10. Q. Lu, J. Liao, S. Liu, X. Wu, L. Liu, L. Xu, “Precise measurement of micro bubble resonator thickness by internal aerostatic pressure sensing,” *Opt. Express*, vol. 24, pp. 20855-20861, 2016.
11. B. Niu, K. Ge, Z. Xu, X. Shi, D. Guo, T. Zhai, “Laser diode pumped polymer lasers with tunable emission based on microfluidic channels,” *Polymers*, vol. 13, pp. 3511, 2021.
12. J. Wu, X. Wang, L. Liao, “Advances in energy-level systems of organic lasers,” *Laser Photonics Rev.*, vol. 16, pp. 2200366, 2022
13. K. Ge, D. Guo, B. Niu, Z. Xu, J. Ruan, T. Zhai, “Pump-controlled RGB single-mode polymer lasers based on a hybrid 2D-3D μ-cavity for temperature sensing,” *Nanophotonics*, vol. 10, pp. 4591-4599, 2021.
14. K. Ge, X. Shi, Z. Xu, et al. “Full-color WGM lasing in nested microcavities,” *Nanoscale*, vol. 13, pp. 10792-10797, 2021.
15. C. Qiao, C. Zhang, Z. Zhou, et al. “A photoisomerization-activated intramolecular charge-transfer process for broadband-tunable single-mode microlasers,” *Angew. Chem. Int. Ed.*,vol. 59, pp. 15992-15996, 2020.
16. W. Talataisong, R. Ismaeel, G. Brambilla, “[A review of microfiber-based temperature sensors](https://www.x-mol.com/paperRedirect/1212919824508592133),” *Sensors*, vol, 18(2), pp. 461, 2018.
17. L. Zhao, Y. Wang, Y. Yuan, *et a*l, “Whispering gallery mode laser based on cholesteric liquid crystal microdroplets as temperature sensor,” *Opt. Commun.*, vol, 402, pp. 181-185, 2017.
18. Z, Liu, L, Lu, Z. Zhu, *et a*l, “Whispering gallery mode temperature sensor of liquid microresonastor,” *Opt. Lett.*, vol. 41, pp. 4649, 2016.
